# Population pharmacokinetic analysis and dosing regimen optimization of penicillin G in patients with infective endocarditis

**DOI:** 10.1186/s40780-016-0043-x

**Published:** 2016-04-05

**Authors:** Toshiaki Komatsu, Takayuki Inomata, Ichiro Watanabe, Masahiro Kobayashi, Hideya Kokubun, Junya Ako, Koichiro Atsuda

**Affiliations:** Department of Pharmacy, Kitasato University Hospital, 1-15-1 Kitasato, Minami-Ku, Sagamihara, Kanagawa 252-0375 Japan; Department of Cardiovascular Medicine, Kitasato University School of Medicine, Sagamihara, Japan

**Keywords:** Penicillin G, Population pharmacokinetics, Dosing optimization, Nonlinear mixed-effects model

## Abstract

**Background:**

This study was designed to evaluate the population pharmacokinetics (popPK) of penicillin G in patients with infective endocarditis and establish a dosage regimen based on pharmacokinetic data and clinical outcome.

**Method:**

Forty-six serum penicillin G samples from 25 individuals were analyzed using a nonlinear mixed-effects model. popPK were estimated using a one-compartment model. We created a receiver operating characteristic (ROC) curve for penicillin G efficacy and the ratio of its minimum concentration (Cmin)/minimum inhibitory concentration (MIC). Simulations were used to optimize the penicillin G dosage regimen using this ratio.

**Result:**

Estimated popPK were: CL (L/h) = 0.21 × creatinine clearance (CLcr) (mL/min), Vd (L) = 28.9. The areas under the ROC curves were 0.87 for clinical efficacy. The cut-off value of penicillin G Cmin/MIC was 60. The continuous administration of 1 million IU penicillin G/h was necessary to achieve a positive outcome for patients with normal renal function (CLcr ≥ 60 mL/min).

**Conclusion:**

Our findings suggest that population-based parameters are useful for evaluating penicillin G pharmacokinetics and that an individualized dosage should be determined based on a described dosage regimen.

## Background

Penicillin G, discovered by Alexander Fleming in 1928, was the first antibiotic to be recognized. Penicillin G is still used widely for the treatment of meningitis, dermatomyositis, and infective endocarditis caused by viridans group streptococci. Nevertheless, infective endocarditis remains a disease with high morbidity and with a mortality rate of 16–37.1 % [1–3]. The 2009 European Society of Cardiology guidelines recommend 4 weeks of monotherapy with penicillin G or ceftriaxone for infective endocarditis caused by susceptible streptococci [4]. The efficacy of penicillin G correlates with the percentage of time above the minimum inhibitory concentration (MIC) (TAM). In general, the therapeutic goal for curing infections caused by gram-positive organisms is an antimicrobial TAM of at least 30 % [5]. At a normal penicillin G dosage of 4 million international units (IU) administered intravenously every 4 h, a TAM of at least 30 % has been reported to result in 100 % penicillin-susceptible (penicillin MIC ≤ 0.125 mg/L) or intermediate penicillin-resistant streptococci (MIC 0.125–2 mg/L). Therefore, we do not believe this parameter is achievable for infective endocarditis [6]. In general, the population pharmacokinetics (popPK) of penicillin G are insufficiently known. Thus, the popPK of penicillin G were analyzed in patients with infective endocarditis. We also determined the relationship between the blood concentrations of penicillin G and clinical outcomes. Furthermore, effective initial dosing regimens for infective endocarditis were determined according to our popPK data.

## Methods

### Data source

Clinical data were collected from patients treated with penicillin G potassium (Meiji Seika Pharma Co. Ltd, Tokyo, Japan) for suspected or documented infective endocarditis at Kitasato University Hospital between January 1997 and April 2013. The collected data included age, gender, height, body weight (BW), alanine aminotransferase (ALT), serum creatinine, creatinine clearance (CLcr), isolated microorganisms, clinical outcome, and serum concentrations of penicillin G. CLcr was estimated from the serum creatinine level by the Cockcroft-Gault method [7]. Failure of penicillin G treatment was defined as persistence of fever and/or bacteremia by the causative pathogen requiring a change in antibiotic therapy, and infection-related mortality within 30 days.

Blood samples were obtained from a brachial vein immediately before and 2 or 3 h after administration of penicillin G for determination of serum penicillin G levels. Human subject procedures were approved by the Ethics Committee of Kitasato University Hospital. In addition, we obtained written informed consent from each patient.

### Assay of penicillin G concentrations

Blood samples were collected using serum-separator tubes, centrifuged immediately at 3000 rpm for 10 min at room temperature and the serum separated. Penicillin G concentrations were measured using minor modifications of a high-performance liquid chromatography (HPLC) method published previously [8]. The mobile phase consisted of acetonitrile and 50 mM potassium phosphate buffer (1:1, v/v), adjusted to pH 5.0 at a flow rate of 0.8 mL/min. Methanol (500 μL) was added to 200 μL of serum. This mixture was vortexed immediately for 30 s and finally centrifuged at 15,000 rpm for 5 min at room temperature. The supernatant was filtered and 20 μL injected into the HPLC. The lower limit of detection of penicillin G was 0.5 μg/mL. The coefficients of variation for both intra- and inter-assay precision were less than 10 %.

### Pharmacokinetic calculations

Data analyses were performed with the nonlinear mixed effects model program (version VI, level 1.0) developed by Beal and Sheiner [9]. A one-compartment pharmacokinetic model was employed using the ADVAN1 and TRANS2 subroutines. The parameters of the structural model were clearance (CL) and volume of distribution (Vd). The first order conditional estimation method was applied for modeling. Inter-individual variability of the parameters was best explained by an exponential error model (Pi = TV(Pi) × exp(ηi)) where Pi indicated an individual value, TV(Pi) was the population value for the parameters described in the equation, and ηi was the random deviation of Pi from TV(Pi). The value of ηi was assumed to be independent and distributed normally with a mean of 0 and a variance of ω^2^. The residual (intra-individual) variability of the parameters was also explained by a proportional error model (Cobs,ij = Cpred,ij × (1 + εij)) where Cobs,ij and Cpred,ij denote the j^th^ observed and predicted concentrations for the i^th^ subject, respectively, and ε is a random intra-individual error that is distributed normally with a mean of 0 and variance σ^2^.

### Covariate analysis

Covariance of the following variables was tested to improve the popPK model: age, serum creatinine, CLcr, BW, ALT, and gender. The influence of continuous covariates on the pharmacokinetic parameter TV(P) was modeled according to the following equations: TV(P) = *θ*p + *θ*c × (covariance), and TV(P) = *θ*p × *θ*c^(covariance)^. Covariance showing a correlation with the pharmacokinetic parameters was introduced into the model. The significance of the influence of covariates was evaluated by a change of −2 log likelihood (the minimum value of the objective function: OBJ). An OBJ decrease of more than 6.63 from the full model (χ^2^: degree of freedom = 1, *P* < 0.01) was considered statistically significant during the forward inclusion process. The full model was structured by incorporating the significant covariates, and the final model was developed by a backward elimination method. When one covariate factor was excluded from the full model, an OBJ that increased more than 6.63 from the full model was considered statistically significant.

### Model evaluation

The adequacy of fitting was examined by plotting the predicted versus the observed concentration of penicillin G, the individual predicted concentration after each Bayesian step versus observed concentration, and the conditional weighted residual concentration versus the predicted one. The accuracy and robustness of the final model were assessed using the bootstrap method [10]. Bootstrap samples were generated by repeated random sampling of the original data set. The size of each bootstrap sample was the same as that of the original sample. Two hundred bootstrap samples were constructed, and the final model was determined by testing these samples repeatedly. The mean parameter estimates obtained from the replicates using normal calculations were compared with those obtained from the original data set.

Target serum concentration of penicillin G in patients with infective endocarditis

The relationship between the penicillin G Cmin/MIC ratio and clinical outcome (clinical failure = 0, clinical success = 1) was analyzed using a logistic regression model. Statistical analyses were performed using JMP statistical software (version 6.03; SAS Institute, Cary, NC, USA). Additionally, receiver operating characteristic (ROC) curve analysis was performed to determine the efficacy of a test by providing information about both sensitivity and specificity at different cut-off points. A two-tailed P value of less than 0.05 was considered significant. The MIC of penicillin G was determined using the MicroScan WalkAway 96 plus (Beckman Coulter, Brea, CA, USA) fully automatic bacterial system that conforms to the micro dilution method according to the Clinical and Laboratory Standards Institute.

### Determination of the dosing regimen

Pharmacokinetic simulations were performed to determine the optimal dosing regimen based on our popPK data. Penicillin G concentrations were simulated for 1000 patients using the final popPK model. For the assessment of efficacy, the percentage of patients that achieved penicillin G Cmin/MIC ratios above 60 was calculated for each data set. For the assessment of safety, the percentage of patients that achieved penicillin G peak concentrations below 100 μg/mL was calculated for each data set. The MIC was fixed at 0.06 and 0.12 mg/L because these MICs are seen frequently in our hospital. Monte Carlo simulation was performed using Crystal Ball 2000 software (Oracle, Redwood Shores, CA, USA).

## Results

Patient characteristics are shown in Table 1. Serum concentrations of penicillin G were measured in a total of 25 patients treated with penicillin G for infective endocarditis. Viridans group streptococci were isolated in 21 of these patients, 15 of whom showed a positive response to penicillin G (success) and 6 showed no response (failure) (Table 2).

**Table 1:** Patient characteristics

Number of Patients	25
Gender (Male:Female)	16:9
Age (year)	54 ± 17^a^ (21–83)^b^
Scr (mg/dL)	0.92 ± 0.56 (0.51–3.26)
CLcr (mL/min)	82.52 ± 33.29 (11–144)
ALT (IU/L)	26.80 ± 26.45 (4–106)
Weight (kg)	55.35 ± 33.31 (33–86.9)
Observation	46
PCG concentraion (μg/mL)	33.2 ± 45.2 (0.5–212.3)

*Scr* serum creatinine, *CLcr* creatinine clearance, *ALT* alanine aminotransferase

^a^Mean ± standerd diviation

^b^Range

**Table 2:** Causative microorganisms

	Total	Penicillin success	Penicillin failure
	*n* = 21	*n* = 15	*n* = 6
Size of vegetation (mm)	11.7 ± 6.0	12.3 ± 6.3	9.8 ± 5.7
PCG MIC	0.11 ± 0.20	0.07 ± 0.02	0.21 ± 0.38
PCG trough serum concentrations (μg/mL)	7.8 ± 9.0	10.1 ± 9.8	2.2 ± 1.2
Viridans group streptococci species level not determined	4	1	3
*streptococcus sanguis*	5	2	3
*streptococcus gordonii*	4	4	0
*streptococcus agalactiae*	2	2	0
*streptococcusmutans*	1	1	0
*streptococcus intermedius*	2	2	0
*streptococcus mitis*	1	1	0
*streptococcus oralis*	1	1	0
*streptococcus constellatus*	1	1	0

**Fig. 1:**
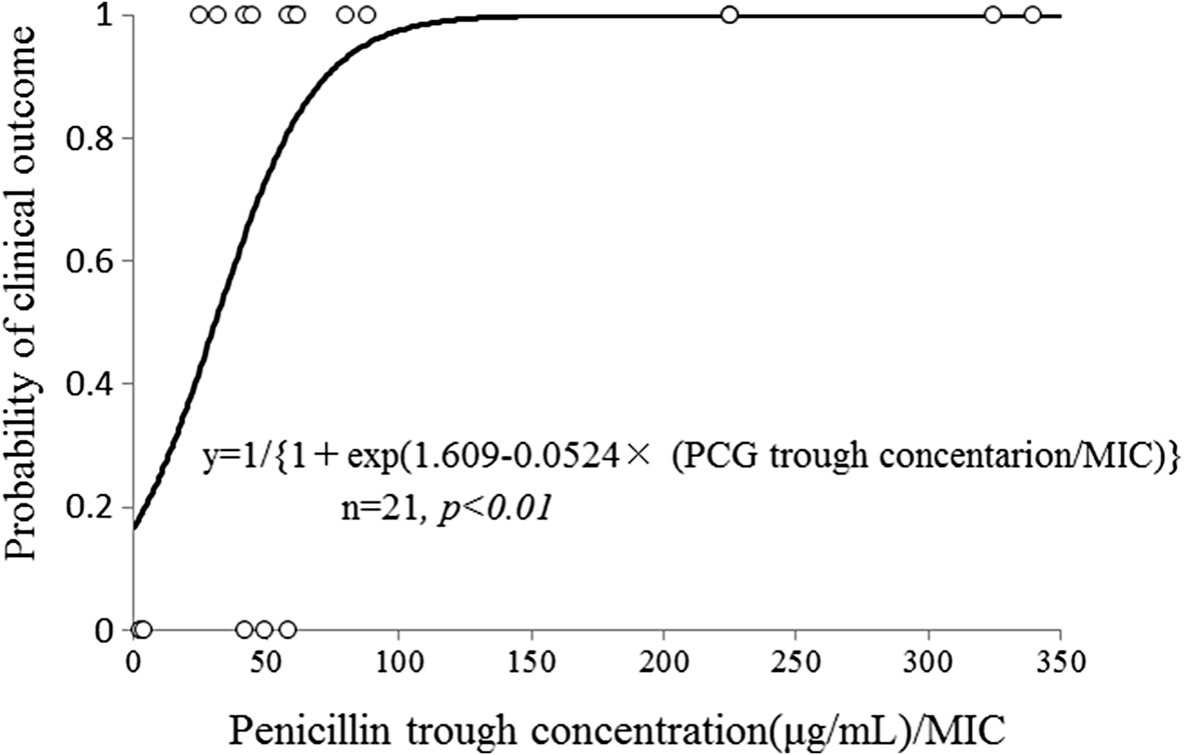
Penicillin G minimum serum concentrations/MIC and logistic regression model for clinical outcomes (failure, 0; success, 1)

**Fig. 2:**
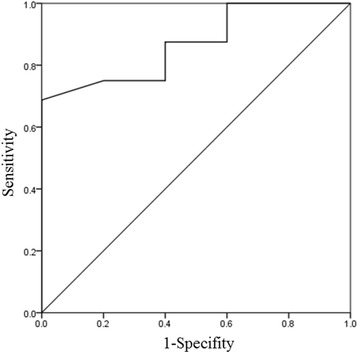
Receiver operating characteristic curve for predicting clinical efficacy using penicillin G minimum serum concentration/MIC

Figure 1 shows the relationship between penicillin G Cmin/MIC and clinical outcome (failure = 0, success = 1). Penicillin G Cmin/MIC was a significant predictor of the following clinical outcome equation: probability of a positive clinical outcome = 1/{1 + exp(1.609–0.0524 × < penicillin G Cmin/MIC>)}. Figure 2 shows the optimal ROC curve for predicting clinical efficacy using the penicillin G Cmin/MIC ratio. The area under the ROC curve was 0.83 and the cut-off value of penicillin G Cmin/MIC for clinical efficacy was 60 (sensitivity 68 %, specificity 100 %).

The results from each covariate model are shown in Table 3. CLcr, serum creatinine, BW, ALT, gender, and age were significant covariates for the CL of penicillin G. During backward deletion from the full model, CLcr remained in the model and caused a significant increase in the OBJ. Table 4 summarizes the popPK estimates from the basic and final models. The final model was: CL (L/h) = 0.21 × CLcr (mL/min), Vd (L) = 28.9. The coefficients of variation of the inter-individual variability (ω^2^) of CL, Vd, and the residual variability (σ^2^) were 28.8, 32.4, and 17.4 %, respectively.

**Table 3:** Hypothesis testing for fixed effect model on penicillin G pharmacokinetics

Fixed model	OBJ	−2.l.l.d	*p*-value
CL = θ1	356.307		
θ1 + θ2 × CLcr	239.004	117.303	<0.001
θ1 + θ2 × 1/sCr	280.388	75.919	<0.001
θ1 + θ2 × BW	323.407	32.9	<0.001
θ1 × θ2^ALT^ (ALT > 40 = 0,ALT < 40 = 1)	337.037	19.27	<0.001
θ1 × θ2^sex^ (Male = 1,Female = 0)	324.973	31.334	<0.001
θ1 × θ2^Age^ (Age > 65 = 1,Age < 64 = 0)	347.699	8.608	<0.005
Vd = θ3			
θ3 + θ4 × BW	356.307	0	N.S.

*N.S* not significant, *CLcr* creatinine clearance, *ALT* alanine aminotransferase, *BW* body weight

**Table 4:** Summary of results for the basic and final population models

	Basic model		Final model	
	CL (L/h) = θ1		CL (L/h) = θ1 × CLcr (mL/min)	
	Vd (L) = θ2		Vd (L) = θ2	
Parameter	Mean	95 % CI	Mean	95 % CI
θ1	8.09	6.75–9.42	0.21	0.173–0.246
θ2	39.1	34.0–44.2	28.9	24.0–33.7
Interindividual variability				
η_CL_	0.765	0.465–1.065	0.0835	0.0292–0.137
ηvd	0.217	0.135–0.298	0.104	0.094–0.115
Residual variability				
ε	0.0643	0.0306–0.0979	0.0304	0.0269–0.0334

η, random variable, which is normally distributed with mean 0 and variance ω2

ε, random error, which is normally distributed with mean 0 and variance σ^2^

*CI* confidence interval, *CLcr* creatinine clearance

**Fig. 3:**
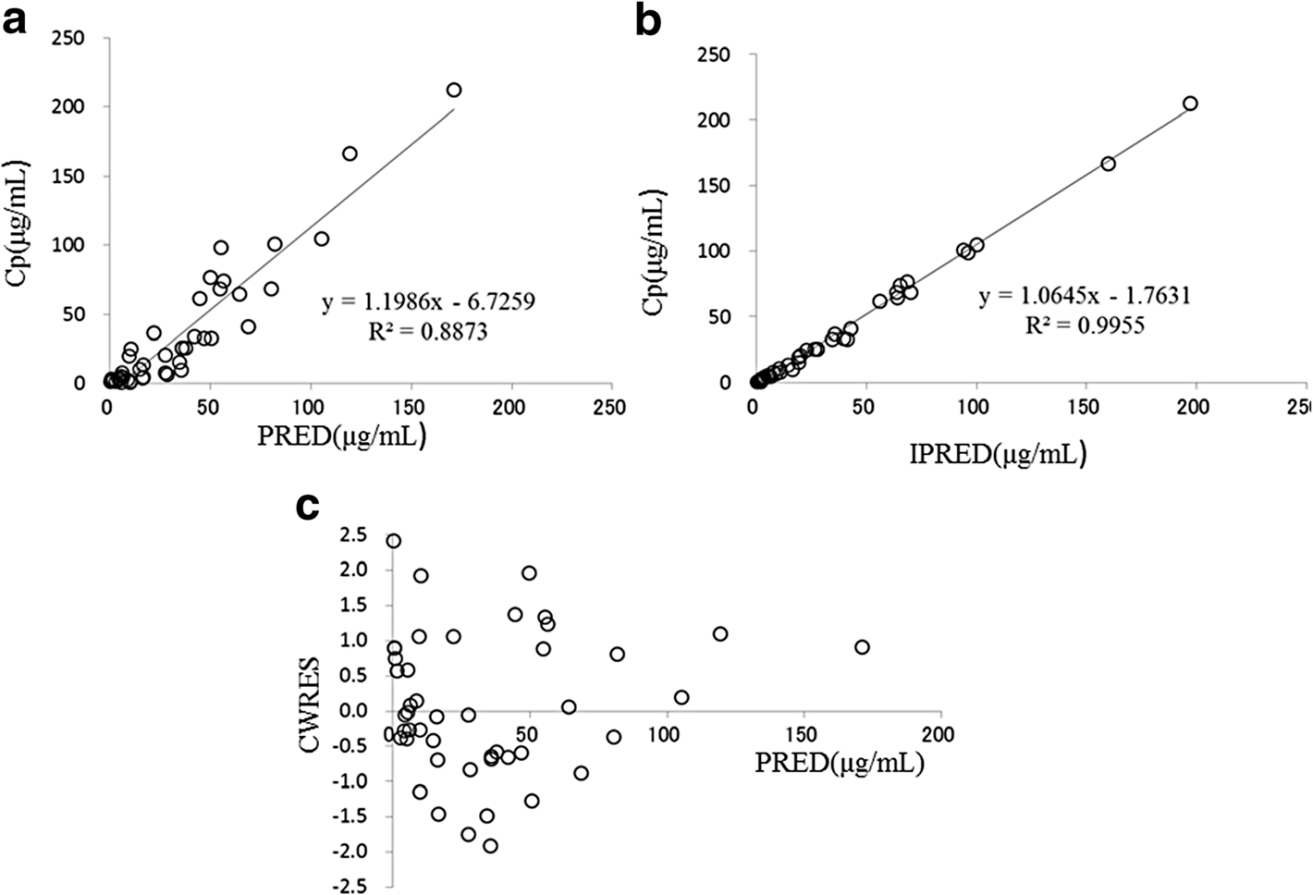
Relationship between observed penicillin serum concentrations (Cp) and predicted concentrations (PRED) (**a**), or individual predicted concentrations after Bayesian fitting (IPRED) (**b**). Scatter plot of conditional weighted residuals (CWRES) versus predicted PRED is presented in (**c**)

Assessment of the predictive performance of the final model is presented in scatter plots of the observed versus the population-predicted concentrations (Fig. 3a), and the individual-predicted concentrations of penicillin G (Fig. 3b). Conditional weighted-residual concentration versus the population predicted concentration is presented in Fig. 3c. These plots are distributed symmetrically around the line of identity, indicating that the model describes the serum concentration of penicillin G adequately.

In the bootstrap analysis of the final model, 158 of 200 bootstraps showed successful results. The values of the parameters used in the final model generated from the bootstrap analysis were similar to those of the developed model (Table 5).

**Table 5:** Final population pharmacokinetic parameters of penicillin G

Parameter	Estimate	RSE (covariance step)	95 % CI (bootstrap method)
Population mean			
CL (L/h) = θ1 × CLcr (mL/min)			
θ1	0.21	8.81 %	0.171–0.249
Vd (L) = θ2			
θ2	28.9	8.58 %	23.4‐34.7
Interindividual variability			
η_CL_	0.0835	33.1 %	0.0171‐0.1397
ηvd	0.104	5.22 %	0.0087–0.1203
Residual variability			
ε	0.0304	5.85 %	0.0265–0.0342

η, random variable, which is normally distributed with mean 0 and variance ω2

ε, random error, which is normally distributed with mean 0 and variance σ^2^

*RSE* relative standard error, *CI* confidence interval, *CLcr* creatinine clearance

**Fig. 4:**
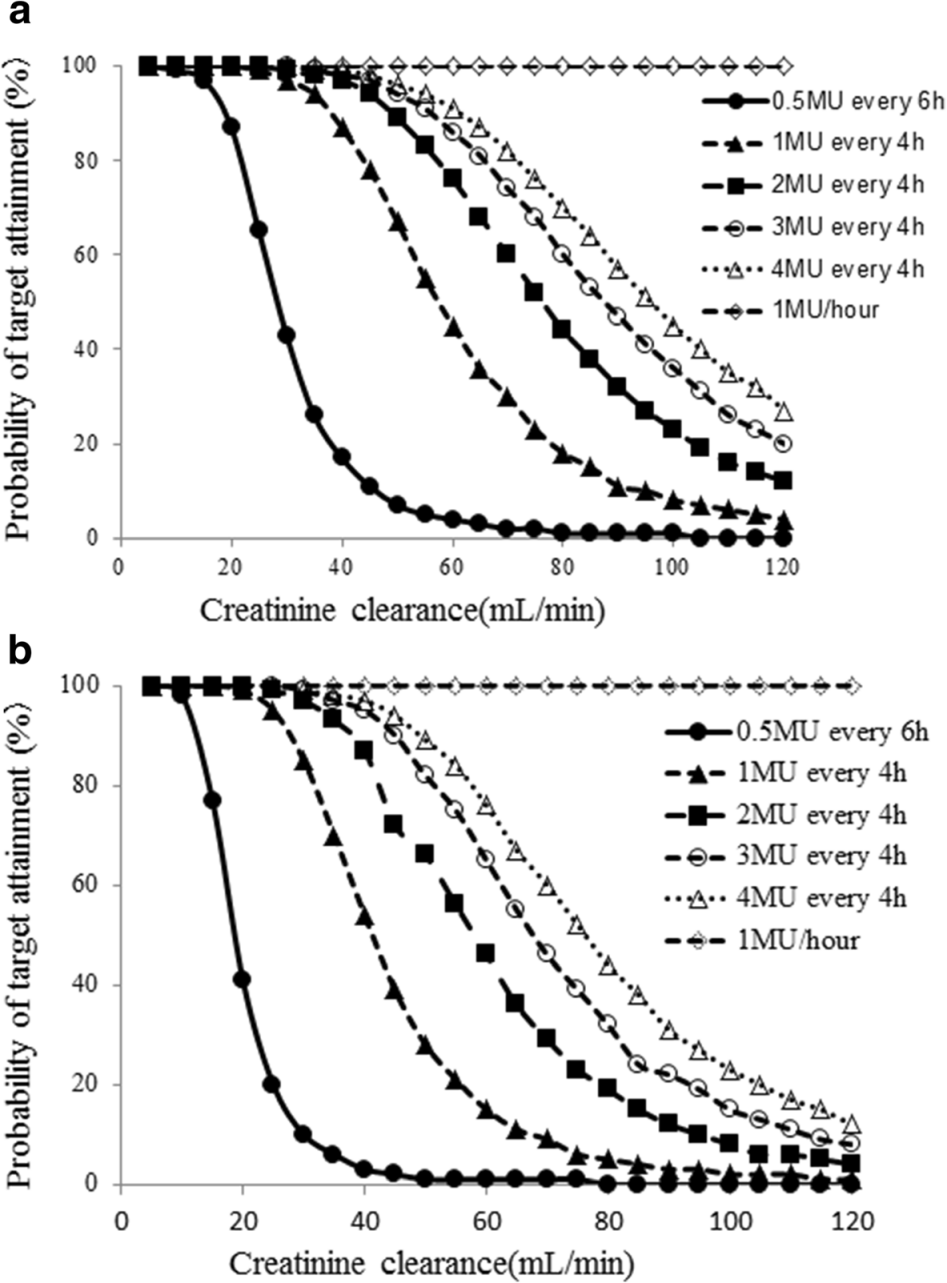
Probability of attaining targets above a penicillin minimum serum concentration/MIC ratio of 60 at CLcr ranging from 5 to 120 with MIC fixed at (**a**) 0.06 or (**b**) 0.12 μg/mL

**Fig. 5:**
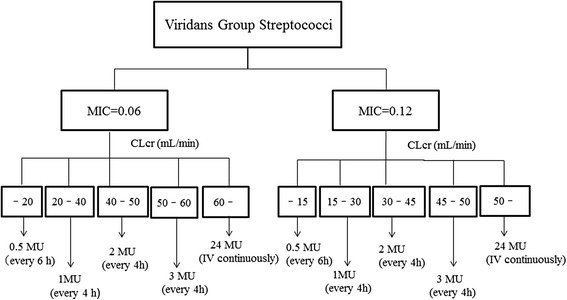
Nomogram for the initial dosage of penicillin against viridans group streptococci

A simulation was performed using the final model to determine the optimal dosing regimen in patients with renal impairment. Penicillin G concentrations were simulated for 1000 patients exhibiting CLcr ranging from 5 to 120 mL/min. The target was a Cmin/MIC value above 60 as estimated by Fig. 2. The simulation was performed for dosages ranging from 0.5 million IU every 6 h to 4 million IU every 4 h, as well as 1 million IU/h (Fig. 4). Figure 5 shows the typical initial dose of penicillin G for MICs of 0.06 and 0.12 μg/mL in patients with various CLcr based on the simulation experiments.

## Discussion

This is the first popPK study of penicillin G that was used to propose a dosing regimen. Although a three-compartment model has been described for penicillin G [11], we used a one-compartment model because our sampling points for serum penicillin G levels were at the trough and 2 or 3 h after administration of penicillin G, and there were few data during the elimination phase. As a result, CLcr was a significant covariant for the systemic clearance of penicillin G that occurs through the kidney [12, 13]. Dittert et al. reported that the Vd for penicillin G was 33 L in healthy people [13]. This value is very close to that obtained in our study (28.9 L). The reported serum half-life of penicillin G in patients with normal renal function ranged from 0.38 to 0.78 h [14, 15]. These values are very similar to our data (0.79 h) calculated by keeping the CLcr fixed at 120 mL/min.

Predictions of the penicillin G concentrations were made with the final regression model. The model exhibited a good linear correlation between observations and the individual predicted concentrations after the Bayesian steps. The final model lacked bias on the relationship between Figs. 3a, b. Specifically, the conditional weighted residuals were acceptable to within three standard deviations, which is generally recognized as the criterion for no selection biases (Fig. 3c). Therefore, we concluded that the final model had a good predictive performance.

The logistic regression model (Fig. 1) and ROC curve (Fig. 2) revealed a significant association between penicillin G Cmin/MIC ratios and the therapeutic effect. The threshold penicillin G Cmin/MIC ratio that led to clinical success was above 60, the cut-off associated with 100 % probability of response to therapy. Figure 4 also showed the probability of success with a penicillin G Cmin/MIC ratio above 60 and a penicillin G peak concentration below 100 μg/mL. At this peak concentration, patients became comatose with continuous muscle jerking [16]. García-Cabrera et al. reported infective endocarditis with a mortality rate of 30 % [17]. Our data showed a 29 % failure rate of penicillin G treatment (6/21). Although there were some patients with a positive response to penicillin G whose penicillin G Cmin/MIC ratios were lower than 60, patients whose values were over 60 all had a positive response.

This regimen was used to determine the typical initial dosage of penicillin G against viridans group streptococci in patients with various CLcr. The continuous administration of 1 million IU/h at a MIC of 0.06 was necessary to achieve therapeutic success against infective endocarditis in patients with normal renal function (CLcr > 60 mL/min). Continuous infusion of penicillin G is recommended by the American Heart Association guidelines [18]. Overall, the method described in this project assured therapeutic efficacy in infective endocarditis with a negligible risk of neurotoxicity [19]. The initial dosage regimen determined using our model will be useful for patients with infective endocarditis caused by *Streptococcus spp.* A prospective study using this regimen will help determine the robustness and reliability of our model.

## Conclusion

This is the first popPK study of penicillin G that was used to propose a dosing regimen. CLcr was a significant covariant for the systemic clearance of penicillin G. Additionally, the results showed that continuous administration of 24 million units of penicillin G over 24 h was necessary to achieve a positive outcome for patients with normal renal function (CLcr ≥ 60). Our findings suggest that population-based parameters are useful for evaluating penicillin G pharmacokinetics and that an individualized dosage should be determined based on a described dosage regimen.

## Abbreviations

ALT: alanine aminotransferase
BW: body weight
CLcr: serum creatinine, creatinine clearance
Cmin: minimum concentration
HPLC: high-performance liquid chromatography
MIC: minimum inhibitory concentration
OBJ: objective function
popPK: population pharmacokinetics
ROC: receiver operating characteristic
TAM: time above the minimum
